# Anthocyanins—Nature’s Bold, Beautiful, and Health-Promoting Colors

**DOI:** 10.3390/foods8110550

**Published:** 2019-11-04

**Authors:** Taylor C. Wallace, M. Monica Giusti

**Affiliations:** 1 Department of Nutrition and Food Studies, George Mason University, Fairfax, VA 22030, USA; 2 Think Healthy Group, Inc., Washington, DC 20001, USA; 3 Department of Food Science & Technology, The Ohio State University, 2015 Fyffe Court, Columbus, OH 43210, USA; giusti.6@osu.edu

**Keywords:** anthocyanin, anthocyanidin, flavonoid, cyanidin, pelargonidin, delphinidin, petunidin, peonidin, malvidin

## Abstract

Anthocyanins are among the most interesting and vigorously studied plant compounds, representing a large class of over 700 polyphenolic pigments within the flavonoid family that exist ubiquitously in the human diet. They are “nature’s colors,” responsible for providing the beautiful red-orange to blue-violet hues present in many leaves, flowers, vegetables, and fruits, especially berries. The beginning of the 21st century has witnessed a renaissance in research activities on anthocyanins in several areas, mainly related to their potential health-promoting properties and their increased use as alternatives to synthetic food colors. There is increasingly convincing scientific evidence that supports both a preventative and therapeutic role of anthocyanins towards certain chronic disease states. Many anthocyanin-based extracts and juice concentrates from crop and/or food processing waste have become commercially available as colorants and/or value-added food ingredients. There is a large and evolving peer-reviewed literature on how anthocyanin chemistry and concentration may affect their coloring properties in food. Equally as important is the food matrix, which can have large impacts on anthocyanin color expression, stability and degradation, particularly regarding the applications of anthocyanins as food colorants and their health-promoting properties. This Special Edition of *Foods*, titled “Anthocyanins in Foods,” presents original research that extends our understanding of these exciting and complex compounds.

Anthocyanins (Greek *anthos* = flower and *kyáneos* = blue) are among the most interesting and vigorously studied plant compounds, representing a large class of over 700 distinct anthocyanin derivatives of 27 aglycons, known as “anthocyanidins.” In addition to their multiple phenyl groups, anthocyanins are rarely found as aglycons. The anthocyanidin typically contains ≥1 sugar moiety commonly conjugated to the C3 hydroxyl group in the C-ring, making them glycosides. With the exception of 3-deoxyanthocyanins, anthocyanins exist almost exclusively in a glycosylated form; their aglycon counterparts are not stable and rarely found in nature. Six anthocyanidins (cyanidin, pelargonidin, delphinidin, petunidin, peonidin, and malvidin) are prominent in nature and represent approximately 90% of all anthocyanins identified to date [1] (Figure 1A). At the primary level, the degree of hydroxylation/methoxylation of the anthocyanidin B-ring and the nature of the sugar and/or acid conjugations have the greatest effect on the color produced by these pigments. An increased number of hydroxyl and/or methoxyl groups on the B-ring of an anthocyanidin results in a bathochromic shift of the visible absorption maximum, which has a bluing effect on the color produced [2]. Substitutions on the R-groups of the B-ring may also affect the stability of the pigment: hydroxylation of the B-ring has been reported to decrease the stability of the anthocyanin, while methoxylation increases the stability. Acylation of the sugar substitutions and/or individual anthocyanidins may also produce a bathochromic (increased wavelength) and/or hyperchromic (increased absorption) shift, altering the spectra of a compound [2,3] (Figure 1B).

Acylation of the anthocyanin, mainly with aromatic acids, can potentially drastically improve its stability through intermolecular/intramolecular co-pigmentation and self-association reactions, making acylated anthocyanins more desirable as food colors [2]. Anthocyanins have also been known to interact with components of the food matrix, including proteins, fat, inorganic salts, metals, and other compounds [4]. There are extremely well-done and comprehensive reviews on the coloring properties of anthocyanins in the peer-reviewed literature [2,3,4,5,6,7,8,9].

The beginning of the 21st century has witnessed a renaissance in research activities on anthocyanins in several areas (Figure 2), mainly related to their potential health-promoting properties and their increased use as alternatives to synthetic food colors.

Daily intake of anthocyanins in the U.S. diet is estimated to be 11.6 ± 1.07 mg/d, with 31 ± 2% of adults ≥20 years having zero intake [10]. The relative bioavailability of anthocyanins has been recently suggested to be between 12–15%, [11,12] as new methodologies such as ^13^C-tracer technologies have helped to identify new anthocyanin metabolites in the bloodstream and urine [10]. There is a reasonable array of emerging epidemiological and clinical evidence that supports the notion that anthocyanins as part of a diet rich in fruits and vegetables have an important role in the prevention of several disease pathologies, most notably cardiovascular disease, type-2 diabetes, certain types of cancers, cognition, and vision. Anthocyanins have been suggested to exert antioxidant, anti-mutagenic, anti-tumoral, anti-inflammatory, and immunomodulatory effects that are associated with maintaining homeostatic balance in the body [13]. A recent systematic review of randomized, controlled trials found that supplementation with purified anthocyanins or anthocyanin-rich extracts improved low-density lipoprotein (LDL) cholesterol among those with hyperlipidemia or elevated biomarkers of metabolic syndrome [14]. Several extremely well-done and comprehensive reviews on the bioavailability and health-promoting properties of anthocyanins have been published in the peer-reviewed literature [15,16,17,18,19,20]. A recent review also highlights new frontiers in polyphenol (including anthocyanin) research in nutrition and health [21]. Clinical evidence indicates that polyphenols may have the potential to reduce the risk of cardiovascular disease [21] through various mechanisms also shown to be exerted by anthocyanins and other subclasses. Inverse relationships between various subclasses polyphenols and type-2 diabetes have also been suggested [21].

In this Special Edition of *Foods*, titled “Anthocyanins in Foods,” we present four original research articles that extend our knowledge of the chemistry, stability, and health-promoting properties of anthocyanins [22,23,24,25]. The Special Edition can be accessed here: https://www.mdpi.com/journal/foods/special_issues/Anthocyanin_Color_additive.

## Figures and Tables

**Figure 1:**
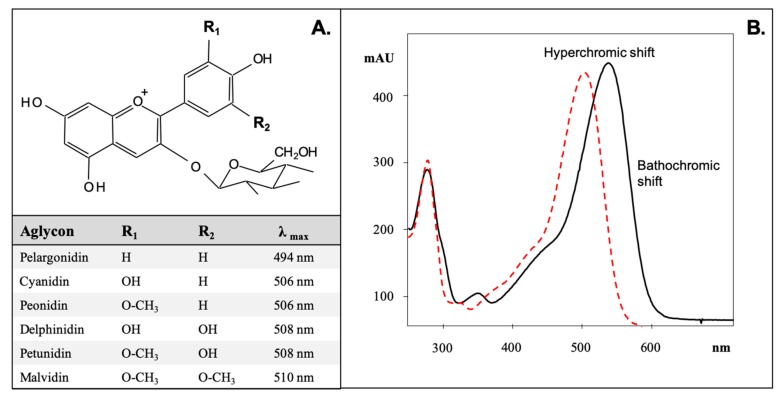
Anthocyanidins commonly found in nature, their B-ring conjugations, and maximum absorbance (**A**). Bathochromic and hyperchromic shifts produced by acylation of anthocyanins (**B**).

**Figure 2:**
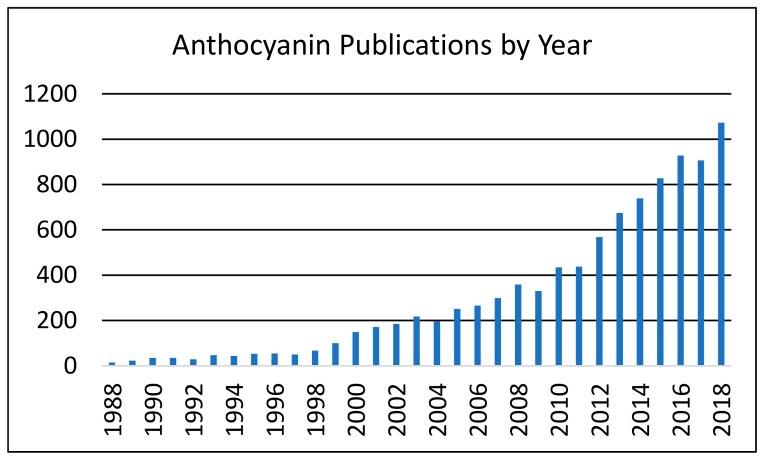
Evolution of the number of papers published in PubMed over the past 30-years (1988 to 2018).

## References

[1] Wallace T.C., Giusti M.M. (2015). Anthocyanins. Adv. Nutr..

[2] Giusti M.M., Wrolstad R.E. (2003). Acylated anthocyanins from edible sources and their applications in food systems. Biochem. Eng. J..

[3] Wrolstad R. (2006). Anthocyanin Pigments-Bioactivity and Coloring Properties. J. Food Sci..

[4] Giusti M.M., Wallace T.C. (2009). Flavonoids as Natural Pigments. Handbook of Natural Colorants.

[5] Wrolstad R.E., Durst R.W., Lee J. (2005). Tracking color and pigment changes in anthocyanin products. Trends Food Sci. Technol..

[6] Castañeda-Ovando A., Pacheco-Hernández M.D.L., Páez-Hernández M.E., Rodríguez J.A., Galán-Vidal C.A. (2009). Chemical studies of anthocyanins: A review. Food Chem..

[7] Zhao C.-L., Yu Y.-Q., Chen Z.-J., Wen G.-S., Wei F.-G., Zheng Q., Wang C.-D., Xiao X.-L. (2017). Stability-increasing effects of anthocyanin glycosyl acylation. Food Chem..

[8] Sigurdson G.T., Tang P., Giusti M.M. (2017). Natural Colorants: Food Colorants from Natural Sources. Annu. Rev. Food Sci. Technol..

[9] Silva V.O., Adilson A.F., António L.M., Frank H.Q. (2016). Chemistry and Photochemistry of Natural Plant Pigments: The Anthocyanins: Chemistry and Photochemistry of Natural Plant Pigments: The Anthocyanins. J. Phys. Org. Chem..

[10] Sebastian R.S., Enns C.W., Goldman J.D., Martin C.L., Steinfeldt L.C., Murayi T., Moshfegh A.J. (2015). A New Database Facilitates Characterization of Flavonoid Intake, Sources, and Positive Associations with Diet Quality among US Adults. J. Nutr..

[11] Czank C., Cassidy A., Zhang Q., Morrison D.J., Preston T., A Kroon P., Botting N.P., Kay C.D. (2013). Human metabolism and elimination of the anthocyanin, cyanidin-3-glucoside: A 13C-tracer study. Am. J. Clin. Nutr..

[12] Ludwig I.A., Mena P., Calani L., Borges G., Pereira-Caro G., Bresciani L., Del Rio D., Lean M.E., Crozier A. (2015). New insights into the bioavailability of red raspberry anthocyanins and ellagitannins. Free. Radic. Boil. Med..

[13] Silva S., Costa E.M., Veiga M., Morais R.M., Calhau C., Pintado M. (2018). Health promoting properties of blueberries: A review. Crit. Rev. Food Sci. Nutr..

[14] Wallace T.C., Slavin M., Frankenfeld C.L. (2016). Systematic Review of Anthocyanins and Markers of Cardiovascular Disease. Nutrients.

[15] Kalt W., McDonald J.E., Vinqvist-Tymchuk M.R., Liu Y., Fillmore S.A.E. (2017). Human anthocyanin bioavailability: Effect of intake duration and dosing. Food Funct..

[16] He J., Giusti M.M. (2010). Anthocyanins: Natural Colorants with Health-Promoting Properties. Annu. Rev. Food Sci. Technol..

[17] Pojer E., Fulvio M., Dan J., Creina S.S. (2013). The Case for Anthocyanin Consumption to Promote Human Health: A Review: Anthocyanins and Human Health…. Compr. Rev. Food Sci. Food Saf..

[18] Tsuda T. (2012). Dietary Anthocyanin-Rich Plants: Biochemical Basis and Recent Progress in Health Benefits Studies. Mol. Nutr. Food Res..

[19] Li D., Wang P., Luo Y., Zhao M., Chen F. (2017). Health Benefits of Anthocyanins and Molecular Mechanisms: Update from Recent Decade. Crit. Rev. Food Sci. Nutr..

[20] Yousuf B., Khalid G., Ali A.W., Preeti S. (2016). Health Benefits of Anthocyanins and Their Encapsulation for Potential Use in Food Systems: A Review. Crit. Rev. Food Sci. Nutr..

[21] Durazzo A., Massimo L., Eliana B.S., Carla C., Elisabetta C., Angelo A.I., Ettore N., Antonello S. (2019). Polyphenols: A Concise Overview on the Chemistry, Occurrence, and Human Health. Phytother. Res..

[22] Chen C.-C., Lin C., Chen M.-H., Chiang P.-Y. (2019). Stability and Quality of Anthocyanin in Purple Sweet Potato Extracts. Foods.

[23] Bariexca T., Ezdebski J., Redan B.W., Vinson J., Bariexca T. (2019). Pure Polyphenols and Cranberry Juice High in Anthocyanins Increase Antioxidant Capacity in Animal Organs. Foods.

[24] Sánchez-Madrigal M.Á., Quintero-Ramos A., Amaya-Guerra C.A., Meléndez-Pizarro C.O., Castillo-Hernández S.L., Aguilera-González C.J. (2019). Effect of Agave Fructans as Carrier on the Encapsulation of Blue Corn Anthocyanins by Spray Drying. Foods.

[25] Vukoja J., Pichler A., Kopjar M. (2019). Stability of Anthocyanins, Phenolics and Color of Tart Cherry Jams. Foiods.

